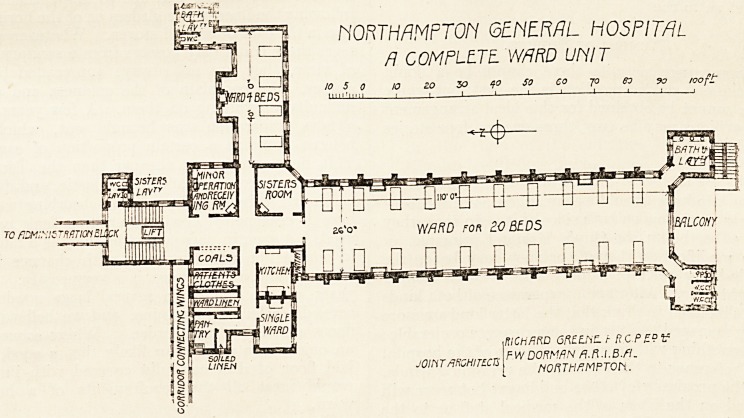# A Complete Ward Unit for a Modern General Hospital

**Published:** 1911-05-06

**Authors:** Richard Greene, Frederick W. Dorman


### Hospital Architecture and Construction.

[Communications on this subject should be marked "Architecture" in the left-hand top corner of the envelope.]

### A COMPLETE WARD UNIT FOR A MODERN GENERAL HOSPITAL.

By RICHARD GREENE, F.R.C.P. Ed., and FREDERICK W. DORMAN, A.R.I.B.A.

### Aspect.

It nearly always happens that the site for a hospital has been obtained by the governors long before the architect is engaged to carry out the work, hence he cannot in all cases choose the best aspect for the pavilions, and has to make the various parts of the buildings fit in with the ground at his disposal as best he can. But when the choice exists, the long axes of the larger wards should invariably run north and south with the free ends of the wards facing the south. This point has been settled with mathematical precision by an American architect, and it is unnecessary further to emphasise the fact that this aspect is the one which ensures the all-important maximum of sunshine in a climate such as that of England. Therefore the architect who has the choice and who neglects this arrangement commits the unpardonable sin in hospital construction.

### Unit.

The ward unit should approach completeness as nearly as possible: That is, it should be in effect a smaller self-contained hospital. The size of the larger wards may be almost anything from ten or twelve beds to twenty-six, or even thirty; but such a number as either of the latter is apt to make the ward unwieldy, especially if the unit include, as it ought to do, a four-bedded ward and a single-bedded ward or two.

### Sanitary Annexes.

The sanitary annexes and bathrooms are best placed at the south end of the ward. It is true that in this position they would obstruct some of the sunshine; but the greater ease in construction, the certainty of obtaining plenty of ventilation around the annexes, and their easy access from the ward make up for the drawback. Furthermore, the space between the annexes can be made into a veranda and used as a lounge by selected patients during the summer months, at any rate, or for the open-air treatment of phthisis. A fire-escape staircase is placed at the end of each ward.

### Wall Space.

The wall space should not be less than ten feet per bed, and it need never exceed eleven feet. The breadth of the ward should be twenty-five or twenty-six feet. This with a ceiling height of eleven feet six inches, or twelve feet, would give a cubic space of about fifteen hundred feet per bed and a superficial area of from one hundred and twenty-five feet to one hundred and forty-three feet. It is certain that the lowest of these measurements should never be greatly reduced; perhaps never reduced at all.

### Appurtenances.

At the north end of the ward on one side we place the sisters' duty-room, and on the other side the ward kitchen. The former should have an inspection window looking into the large ward, and a similar window commanding the four-bedded ward. The latter is provided with a large bay-window and a fireplace. A bathroom and closet are attached to the ward. The kitchen has a hatch for the direct delivery of special articles of diet to the large ward. On the north side of the passage leading to the fourbedded ward is a room which could be used as a minor operation room, or for special examinations, or as a retiring-room for the honorary physician or surgeon. On the opposite side of the main passage is the store for ward coal; then the store for patients' clothing, the ward-linen store, and the shoot for soiled linen.

Next to the ward kitchen is the single-bedded room. This room has two windows on its south side and one on its north side, and there is also a large fanlight over the door. By these means the most efficient cross-ventilation can be obtained—and it is just as important, or more important, that these rooms should be cross-ventilated as the larger ones. The student of hospital architecture is often aghast at the insufficient ventilation of single-bedded rooms, and even of small wards.

Further north is the staircase of the block, with its lift; and opening from the landing is the nurses' lavatory. Running east and west is the corridor connecting the various blocks; and from the staircase is the corridor connecting the pavilion with the administrative block. Here the ward unit ends; but there are other points which may be referred to: First as to the

### Number of Stories.

Where space permits there cannot be a doubt that the one-storied pavilion is the best, but where space cannot be obtained for this ideal arrangement the pavilions may be two, three or even four stories in height.

### The Windows.

The windows should be arranged so that every bed has a window on both sides of it. In form they may either be on the Guy's Hospital principle, or some modification thereof, or they may be on the double-hung sash principle, with a large hopper fanlight over. After some experience of both kinds we are inclined to think that the latter kind are more easily managed and are in some respects preferable; but something will depend on the site of the hospital with reference to the climate, the prevailing winds, and the proximity of other buildings; but more will depend on the ideas of the medical staff as to the necessity of providing the minimum of three or four thousand cubic feet of fresh air per hour per patient. Where this is insisted on the Guy's window will be found to be the easiest and least objectionable method of obtaining it. Whichever form be adopted the heads of the windows should be as near the ceiling as it is possible to get them.

### Fireplaces.

The fireplaces may be either in the external walls, or central, partly-enclosed stoves may be used. The latter is, at present, the favourite system; but the stoves tend to disfigure the ward and they take up a considerable amount of valuable floor-space. The greatest objection, which they share in common with all other enclosed stoves and with hot-water or steam radiators, is that they warm the air which is breathed by the patients; whereas the old, time-honoured open fireplaces do not. The rays of heat from an open fire pass through the air without warming it, thus resembling the sun's rays, and open fires are therefore the only perfectly hygienic means of warming hospital wards. A few hot-water radiators may be placed in the large wards, but their use should be very jealously guarded by the medical staff, and they should only be brought into play when the weather is exceptionally cold. As a matter of fact, patients when in bed and sufficiently supplied with blankets rarely complain of cold. The success of the open-air treatment of phthisis has proved this. In any case, to allow a patient to breathe the air which is warming his body savours of the disgusting, and no hospital which habitually uses artificially-warmed air can hope to obtain the best results in the treatment of disease.

### The Floors.

Terrazzo or granolithic floors are now much in vogue, and beyond doubt a smooth, non-absorbing surface is thereby obtained; but unless laid with extreme care cracks are apt to show themselves following the lines of the girders or of the horizontal flue in the case of central stoves. Whatever advantages these floors may possess from a sanitary point of view, they are not always appreciated by the nurses, who complain of the coldness and of the difficulty of walking on them. A few years since a new preparation was brought out, which, we believe, contains a certain proportion of sawdust. This floor-covering is cheaper than the Terrazzo, and it is also warmer and more elastic, and if it can be shown to be durable and non-absorbing we have here a reasonably good material. Teak may be used, but even in narrow widths there is a tendency to contraction and expansion with changes in the weather, and, furthermore, it is very costly. Many years ago we laid down several large wards with carefully selected pitch-pine, and we believe these floors are still in good order. Where economy is an object it is more than likely that a good, well-laid floor of deal carefully covered with linoleum would meet all the requirements of a hospital ward.

The accompanying plan is substantially a copy of a ward unit from the Northampton Hospital, which was erected a few years since by the authors of this paper, and which contains one hundred and sixty beds.

## Figures and Tables

**Figure f1:**